# Supplementary material for: The RLR-MAVS-IRF3 axis activates the IFN pathway to restrict Tonate virus infection
Source: PLoS Negl Trop Dis. 2026 Oct 16;20(10):e0014559. doi: 10.1371/journal.pntd.0014559 (PMC13712696; doi:10.1371/journal.pntd.0014559)
Supplement: Supplementary file 1 [file pntd.0014559.s005.pdf]

Revision. Manuscript PNTD-D-26-01338

This study from *Labadie* and colleagues explores an important question in alphavirus biology: how Tonate virus (TONV), a neglected encephalitic alphavirus of the Venezuelan equine encephalitis complex, is sensed in human cells and how that sensing drives type I interferon production and downstream responses. The authors use a strong set of experimental approaches, including infection of human cell lines, genetic knockouts of RIG-I, MDA5, MAVS, and IRF3, and complementary readouts of viral replication, IFN-I induction, and ISG expression. While some weaknesses have been identified (see point-by-point comments), this study provides solid evidence that the RIG-I/MDA5–MAVS–IRF3 axis is a major pathway of TONV restriction in human cells. The work is well written, clearly articulated, and fills a genuine gap, as this question has not been specifically examined for TONV.

Comments:

Fig 3A. It is intriguing that MAVS protein levels appear less intense in WT cells than in the other KO lines (except the MAVS KO). In addition, residual MAVS signal is still detected by Western blot in the MAVS KO line and, by eye, does not differ clearly from WT. Please add densitometric quantification and discuss in the text why residual MAVS signal may be detected in the KO line.

Fig. 3B-D show differences in viral replication, IFN-I induction, and downstream responses between IRF3-KO and MAVS-KO cells, suggesting additional sensing pathways contributing to IFN-I induction. TLR3-TRIF signaling can activate IRF3 and has been implicated in alphavirus infection (PMID 22761364; PMID 27864552). Additional IRF3 activation through this pathway could explain the IRF3-KO versus MAVS-KO difference and is consistent with MAVS-IRF3 not being the unique or major pathway implicated on TONV-induced IFN-I production. The study would be strengthened by testing the contribution of TLR3 or, at minimum, discussing this additional possibility.

Fig. S2B. In the Results, the authors state that “We also confirm that IFN pretreatment increases ISG levels in both cell lines”. This statement is not supported for A549 cells. Also, add statistical analysis for this experiment.

Minor:

- **Fig. 1B.** For clarity, specify the cell line used. This is not apparent from either the text or the figure.
- **Fig. 1D.** The nature of the IFN-I reporter assay is unclear. The manuscript would benefit from one sentence in the Results stating whether this is a reporter-cell assay or a direct measurement of interferon (e.g., ELISA). This information is already in the Materials and Methods, but a brief clarification in the Results would improve readability.
- **Fig. 2**. It is not clear from the text as written that the KO lines were generated in the glioblastoma cell line. Please clarify in the result description in which cell types the KO were made.
