# Supplementary material for: The RLR-MAVS-IRF3 axis activates the IFN pathway to restrict Tonate virus infection
Source: PLoS Negl Trop Dis. 2026 Oct 16;20(10):e0014559. doi: 10.1371/journal.pntd.0014559 (PMC13712696; doi:10.1371/journal.pntd.0014559)
Supplement: Supplementary file 2 [file pntd.0014559.s007.docx]

**The RLR-MAVS-IRF3 axis activates the IFN pathway to restrict Tonate virus (TONV) infection**

Thomas Labadie^1^*, Roger J. Eloiflin^1^*, Zoé Denis^1^, Marie Motos^2^, Joanna Re^1^, Moritz Schussler^1^, Morgane Chemarin^1^, Isabelle Moltini-Conclois^2,3^, Valerie Courgnaud^1^, Dorothée Missé^2,3^, Nadine Laguette^1+^, Karim Majzoub^1+^

1- Institut de Génétique Moléculaire de Montpellier (IGMM), Univ Montpellier, CNRS, Montpellier, France

2- MIVEGEC, Univ. Montpellier, CNRS, IRD, Montpellier, France.

3- Department of Medical Technology, LMI PRESTO, Faculty of Associated Medical Sciences, Chiang Mai University, Chiangmai, Thailand.

*These authors contributed equally to this work

^+^ Corresponding authors: [nadine.laguette@igmm.cnrs.fr](mailto:nadine.laguette@igmm.cnrs.fr) , [karim.majzoub@igmm.cnrs.fr](mailto:karim.majzoub@igmm.cnrs.fr)

**JOURNAL REQUIREMENTS**

1) Please ensure that the CRediT author contributions listed for every co-author are completed accurately and in full.
At this stage, the following Authors/Authors require contributions: Thomas Labadie, Roger Eloiflin, Zoe Denis, Marie Motos, Joanna Re, Moritz Schussler, Morgane Chemarin, Isabelle Moltini-Conclois, Valerie Courgnaud, Dorothée Missé, Nadine Laguette, and Karim Majzoub. Please ensure that the full contributions of each author are acknowledged in the "Add/Edit/Remove Authors" section of our submission form.
The list of CRediT author contributions may be found here: <https://journals.plos.org/plosntds/s/authorship#loc-author-contributions>

Author Contributions has been updated as requested in the new version of the manuscript.

2) Please provide an Author Summary. This should appear in your manuscript between the Abstract (if applicable) and the Introduction, and should be 150-200 words long. The aim should be to make your findings accessible to a wide audience that includes both scientists and non-scientists. Sample summaries can be found on our website under Submission Guidelines: <https://journals.plos.org/plosntds/s/submission-guidelines#loc-parts-of-a-submission>

The Author Summary has now been included in the main text.

3) We do not publish any copyright or trademark symbols that usually accompany proprietary names, eg (c), (R), or TM (e.g. next to drug or reagent names). Therefore, please remove all instances of trademark/copyright symbols throughout the text, including:
- TM on pages: 19, and 20.

This has now been removed from the text

4) Please upload all main figures as separate Figure files in .tif or .eps format. For more information about how to convert and format your figure files please see our guidelines: <https://journals.plos.org/plosntds/s/figures>

The main figures have been uploaded as separate figure files in .tif format

5) We have noticed that you have uploaded Supporting Information files, but you have not included a list of legends. Please add a full list of legends for your Supporting Information files after the references list.

We are sorry for this omission. The list of legends has now been included in the manuscript

6) In the online submission form, you indicated that All data are available in the manuscript and upon request. All PLOS journals now require all data underlying the findings described in their manuscript to be freely available to other researchers, either
1. In a public repository
2. Within the manuscript itself
3. Uploaded as supplementary information.
This policy applies to all data except where public deposition would breach compliance with the protocol approved by your research ethics board. If your data cannot be made publicly available for ethical or legal reasons (e.g., public availability would compromise patient privacy), please explain your reasons by return email and your exemption request will be escalated to the editor for approval. Your exemption request will be handled independently and will not hold up the peer review process, but will need to be resolved should your manuscript be accepted for publication. One of the Editorial team will then be in touch if there are any issues.

Raw data produced in this work are now included in the manuscript supplementary information. These include qPCR raw Ct value data as well as microcopy images and quantification and uncropped and unedited Western blot images.

7) Please amend your detailed Financial Disclosure statement. This is published with the article. It must therefore be completed in full sentences and contain the exact wording you wish to be published.
i) State what role the funders took in the study. If the funders had no role in your study, please state: "The funders had no role in study design, data collection and analysis, decision to publish, or preparation of the manuscript."
ii) If any authors received a salary from any of your funders, please state which authors and which funders..

If you did not receive any funding for this study, please simply state: The authors received no specific funding for this work.

Funding: This work was co-funded by the European Union (ERC CoG, SENTINEL 101087092 to NL and ERC Stg DELV 101039538 to KM). The Agence National de la Recherche [AlphA to NL, DeltADAPT to KM and TL and ANR-10-LABX-25-01 to DM]. The funders had no role in study design, data collection and analysis, decision to publish, or preparation of the manuscript.

-8) Your current Financial Disclosure states, "This work was co-funded by the European Union (ERC CoG, SENTINEL 101087092 to NL and ERC Stg DELV 101039538 to KM). The Agence National de la Recherche [AlphA to NL, DeltADAPT to KM and and ANR-10-LABX-25-01 to DM] and the Agence Nationale de Recherche sur le SIDA et les Hépatites virales (ANRS)

[ECTZ117448 to NL and RJE].". However, your funding information on the submission form not indicates "ECTZ117448 ". Please, indicate by return email the full and correct funding information for your study and confirm the order in which funding contributions should appear. Please be sure to indicate whether the funders played any role in the study design, data collection and analysis, decision to publish, or preparation of the manuscript.

Please refer to our answer above.

9) PLOS now requires that authors provide the original uncropped and unadjusted images underlying all blot or gel results reported in a submission's figures or Supporting Information files. This policy and the journal's other requirements for blot/gel reporting and figure preparation are described in detail at https://journals.plos.org/plosone/s/figures#loc-blot-and-gel-reporting-requirements and <https://journals.plos.org/plosone/s/figures#loc-preparing-figures-from-image-files>.

When you submit your revised manuscript, please ensure that your figures adhere fully to these guidelines and provide the original underlying images for all blot or gel data reported in your submission. See the following link for instructions on providing the original image data:

<https://journals.plos.org/plosone/s/figures#loc-original-images-for-blots-and-gels>

In your cover letter, please note whether your blot/gel image data are in Supporting Information or posted at a public data repository, provide the repository URL if relevant, and provide specific details as to which raw blot/gel images, if any, are not available. Email us at customercare@plos.org if you have any questions.

Original blot images are now provided in the supplementary information files (Figure_S4_raw_images.tiff)

10) Please send a completed 'Competing Interests' statement, including
any COIs declared by your co-authors. If you have no competing interests
to declare, please state "The authors have declared that no competing
interests exist". Otherwise please declare all competing interests
beginning with the statement "I have read the journal's policy and the
authors of this manuscript have the following competing interests:".

The authors have declared that no competing interests exist.

**RESPONSE TO REVIEWERS**

We thank the reviewers for their very positive feedback and their constructive comments. We also apologize for submitting the manuscript without the figure legends, which we now included in the new version. Please find a point-by-point response to the reviewer’s comments below.

**METHODS**

**Reviewer #1**: The study by Labadie and colleagues investigates how Tonate virus (TONV), a neglected encephalitic alphavirus of the Venezuelan equine encephalitis complex, is sensed in human cell lines and how this sensing induces both IFN-I production and subsequent IFN-I responses. Although both encephalitic and arthritogenic alphaviruses are known to trigger IFN-I responses, this has not been specifically examined for TONV. The manuscript is well written and clearly articulated.

**Reviewer #2**: The study objectives are articulated to describe the TONV innate immune sensing of human cells.

The study design is appropriate to address the objectives.

The required sample size for the in vitro assays is sufficient.

The study contained statistical analysis that can support the observations.

**Reviewer #3:** All the components mentioned above, including the objectives, methods, and statistical analyses, are well described.

**RESULTS**

**Reviewer #1**: Results: The results and figures are appropriate.

Line-by-line comments:

Fig 3A. It is intriguing that MAVS protein levels appear less intense in WT cells than in the other KO lines (except the MAVS KO). In addition, residual MAVS signal is still detected by Western blot in the MAVS KO line and, by eye, does not differ clearly from WT. Please add densitometric quantification and discuss in the text why residual MAVS signal may be detected in the KO line.

Our MAVS KO cells were obtained by transduction with a lentiviral vector expressing CRISPR guide RNA and the *Sp*Cas9 protein, followed by antibiotic selection. This could explain the residual MAVS band observed in the KO cells as these cells are a pooled population and not clonal. Nevertheless, we added a densitometric quantification (Sup. Figure 3A) of our western blot data as requested, that clearly shows that after normalization with GAPDH loading control signal, the residual MAVS protein in the MAVS KO cells is at least two-fold less abundant than in WT cells.

Regarding MAVS levels in RIG-I, MDA5 and IRF3 KO cell lines, our blots indeed show an increase in MAVS quantity when compared to WT cells (Sup. Figure 3A). A plausible scenario that can explain this discrepancy stems from the observation that activation of RIG-I and MDA5 can trigger MAVS ubiquitination marking it for proteasomal or autophagic degradation (PMID: 22626058, PMID: 31304625). In the absence of RIG-I and MDA5 in the KOs the turnover of MAVS protein is slowed which might contribute to a higher accumulation in these KOs. More generally, it is admitted CRISPR-generated mutations can cause a transcriptional adaptation, where mutant transcript degradation can induce compensatory gene expression, upregulating genes involved in the same pathway (PMID: 30944477; review PMID: 40499457).

Importantly, both these scenarios do not alter the validity or interpretation of our data. RIG-I deficiency has been reported to impair viral-induced activation of MAVS rather than promoting its activation (PMID: 27992402). Therefore, an increase in MAVS protein abundance in RLR KO cells should not be interpreted as evidence of enhanced MAVS activation or signaling.

Fig. 3B-D show differences in viral replication, IFN-I induction, and downstream responses

between IRF3-KO and MAVS-KO cells, suggesting additional sensing pathways contributing to IFN-I induction. TLR3-TRIF signaling can activate IRF3 and has been implicated in alphavirus infection (PMID 22761364; PMID 27864552). Additional IRF3 activation through this pathway could explain the IRF3-KO versus MAVS-KO difference and is consistent with MAVS-IRF3 not being the unique or major pathway implicated on TONV-induced IFN-I production. The study would be strengthened by testing the contribution of TLR3 or, at minimum, discussing this additional possibility.

The reviewer raised a very valid and interesting point regarding the difference in our results with the MAVS KO versus IRF3 KO cells. Indeed, we cannot exclude the possibility that Tonate viral RNA is also sensed by a MAVS-independent and IRF3- or NF-κB- dependent pathway, such as the TLR3/TRIF signaling pathway. Another possibility that could explain this difference is the residual levels of MAVS in our MAVS KO cells. MAVS KO with residual MAVS levels (Sup. Fig 3A) could contribute a residual activation of the pathway and higher ISGs stimulation compared with the IRF3 KO cells where the KO was much cleaner (Fig3A and Sup. Fig 3A). We agree with the reviewer that this point should absolutely be discussed and we now mention the possibility of another pathway sensing TONV in human cells, involving TLR3-TRIF, as shown with other alphaviruses (e.g. CHIKV). We thanks the reviewer for providing the references that we now included in the discussion and we mention that the possibility of another sensing pathway should be addressed in future research on TONV.

Fig. S2B. In the Results, the authors state that “We also confirm that IFN pretreatment increases ISG levels in both cell lines”. This statement is not supported for A549 cells. Also, add statistical analysis for this experiment.

We agree with the reviewer, when statistical analysis was performed on A549 conditions, IFN treatment did not significantly upregulate MX1 ISG mRNA levels (FigS2B). We now provide statistical analysis of the data in figure S2B. For T98G treated cells, we obtained a statistically significant increase after IFN treatment (22-fold increase in 1000 U/mL IFN treated cells compared with non-treated, p-value < 0.05, N=3, student t-test) supporting a strong induction of Mx1. The same analysis for the A459 treated cells resulted in non-significant differences observed but yet a modest Mx1 induction at higher IFN doses (3-fold increase in 1000 U/mL IFN treated cells compared with non-treated, p-value > 0.05, n=3, student t-test). This discrepancy could be caused by a cell-line dependent timing differences in respons to IFN-I treatment. Nevertheless, the inhibition of Tonate virus replication after IFN-I treatment in in both cell lines (Figure 2) point to an efficient functional response to IGN-I in A549 cells. To be rigorous we now replace the statement “We also confirm that IFN pretreatment increases ISG levels in both cell lines” to “We also confirm that IFN pretreatment increases ISG levels in T98G cell line”.

**Reviewer #2:** The results presented require clear description and organization. Several sentences are redundant, and some of the sentences and citations can be placed in the Introduction and Discussion sections.

We thank the reviewer for his comments. We now remove redundancy in the text as much as possible to improve our manuscript.

Further description of the results in the text is required. For example: in case of any significant differences (p-values) in the ISG (MX1) levels in both cell lines during the IFN-1 treatment, TONV RNA loads in the absence of IRF3 at different MOI. It would be interesting to discuss the low IFN-β production compared to WT cells upon infection at low MOI (E.g., 0.001).

In line with comment from reviewer #1, we now comment Mx1 levels in both cell lines with statistical analysis, as well as TONV RNA loads in different conditions of MOIs. We also discuss the low IFN- β production at low MOIs as requested.

Please include the legends for all the manuscript figures, as they were not found in the submission documentation. Figure legends are critical for revising the figures.

We are sorry for this omission, figure legends are now provided.

Provide a color image for Figure #4, as no differences are readily visible between the non-infected and infected T98G cells.

We replaced the black and white images by color images, and replaced images for both Non-Infected and WT cells. Positive J2 staining in non-infected cells reflects the presence of dsRNA background from non-viral origin (likely from mitochondrial origin) and is expected to some level (PMID: 34815573).

**Reviewer #3:** Analyses and results are clearly presented.

**CONCLUSIONS**

**Reviewer #1**: - The conclusions are supported by the data.

- The study would be strengthened by discussing the contribution of other sensors such as TLR3.

As requested, we now discuss the putative contribution of TLR3-TRIF in TONV sensing with relevant references.

- Limitations are described appropriately

- The authors discuss both the advance in knowledge as well as public health relevance.

**Reviewer #2**: Conclusions are partially supported by the data presented. The authors described limitations of the study, such as the use of two immortalized human cell lines, the exact viral RNA species recognized during TONV infection, and the contribution of the two RLRs over time.

Yes, the authors discussed how this model can be helpful for testing antivirals.

Yes, studying TONV innate immune sensing in vitro has relevance to public health.

**Reviewer #3**: The conclusions are well supported by the data presented, and the limitations of the study are appropriately acknowledged.

**Editorial and Data Presentation Modifications?**

**Reviewer #1**: Fig. 1B. For clarity, specify the cell line used. This is not apparent from either the text or the figure.

In figure 1B, the cell line is T98G and now mentioned in the title of each subplot and figure legends.

Fig. 1D. The nature of the IFN-I reporter assay is unclear. The manuscript would benefit from one sentence in the Results stating whether this is a reporter-cell assay or a direct measurement of interferon (e.g., ELISA). This information is already in the Materials and Methods, but a brief clarification in the Results would improve readability.

Thanks for this point, we now clarify the IFN-I assay that we perform in the results section.

Fig. 2. It is not clear from the text as written that the KO lines were generated in the glioblastoma cell line. Please clarify in the result. description in which cell types the KO were made.

We thank the reviewer, indeed it was kind of unclear but we now clarify that the KOs were generated in the T98G cell line in the result section.

**Reviewer #2**: (No Response)

**Reviewer #3:** (No Response)

**GENERAL COMMENTS**

**Reviewer #2:**

Tonate virus (TONV) is a neglected arbovirus of the Venezuelan equine encephalitis complex associated with febrile illness and severe disease. However, TONV epidemiology and diagnostics are under the umbrella of dengue disease in the Americas. The study aimed to

describe the interaction between TONV and the IFN-I system in two human cell lines. T98G and A549 cells derived from human glioblastoma and lung carcinoma, respectively, were susceptible to TONV infection at different multiplicities of infection (MOI). TONV infection elicited an IFN-I response in both human cell lines. A further treatment with exogenous IFN-I inhibited TONV infectivity and viral RNA, with increased levels of certain Interferon-stimulated genes. Furthermore, TONV infection led to the accumulation of cytosolic double-stranded (ds)RNA. In vitro depletion of cellular components upstream of the IFN-I generation (RIG-I, MDA5, MAVS, but mainly IRF-3) increased viral RNA and dsRNA-positive cells. The study indicated the RIG-I/MDA5-MAVS-IRF3 axis as a major sensing pathway during TONV infection, and described for the first time a molecular characterization of TONV innate immune sensing in human cells. However, the manuscript requires more attention in the methods and results sections for further publication consideration.

Comments

• Include line numbers throughout the manuscript

This is now included

• Include Figure legends

This is now included, again we apologize for sending this first version of our manuscript without the figure legends.

• Suggest following the research article structure: Introduction, followed by Materials and Methods, Results, and Discussion/Conclusions.

We now moved the materials and methods section before the results section

• Figure #4. Clarify the % difference in positive cells between infected and non-infected WT cells.

Positive J2 staining in non-infected cells reflects the presence of dsRNA background from non-viral origin (likely from mitochondrial origin) and is expected to some level (PMID: 34815573).

• Support the Discussion section by citing Figures

We now cite figures in the discussion

• Provide the passage history of the TONV CaAn 410d straine

This is now mentioned in the methods section:

Tonate virus CaAn 410d strain (CRORA collection, Institut Pasteur, Paris) was isolated from a Psarocolius decumanus (Crested oropendola) in French Guiana, following passage in newborn mice in 1973. The virus was then retrieved and passaged nine times on VERO cells.

.

• Justify the use of two different housekeeping genes (18S and GAPDH)

This work is a collaboration between two laboratories with different routines, which is reflected by the use of different housekeeping genes. 18S ribosomal RNA and GAPDH genes are both widely used as internal control for qPCR experiments for their abundance and stability (PMID: 31882847).

**Reviewer #3**: This is a well-written and well-structured manuscript describing the mechanisms underlying type I IFN inhibition following TONV infection in immune cell lines in vitro. Although the experiments were performed using immortalized cell lines, the authors conducted a comprehensive series of important experiments investigating critical components of the type I interferon pathway in immune cells.

Minor comment

As all experiments were performed using immortalized cell lines, I suggest revising the statement in the abstract that says, “…we investigated the interaction between TONV and the type I interferon (IFN-I) system in human cells.” This statement may be too broad, given that the study was conducted exclusively in immortalized cell lines. The authors may wish to specify this experimental context more clearly.

We agree, we now mention the cell lines used in the abstract.
