# Supplementary material for: The RLR-MAVS-IRF3 axis activates the IFN pathway to restrict Tonate virus infection
Source: PLoS Negl Trop Dis. 2026 Oct 16;20(10):e0014559. doi: 10.1371/journal.pntd.0014559 (PMC13712696; doi:10.1371/journal.pntd.0014559)
Supplement: Peer review history

# Peer review: Decision Letter 0

**DOI:** 10.1371/journal.pntd.0014559.r001

**Authors:** Doug Brackney, David Safronetz

21 Sep 2026

Response to ReviewersRevised Manuscript with Track ChangesManuscript'.

Shaden Kamhawi

co-Editor-in-Chief

PLOS Neglected Tropical Diseases

orcid.org/0000-0003-4304-636XX

Paul Brindley

co-Editor-in-Chief

PLOS Neglected Tropical Diseases

orcid.org/0000-0003-1765-0002

**Journal Requirements:**

1) Please ensure that the CRediT author contributions listed for every co-author are completed accurately and in full.

At this stage, the following Authors/Authors require contributions: Thomas Labadie, Roger Eloiflin, Zoe Denis, Marie Motos, Joanna Re, Moritz Schussler, Morgane Chemarin, Isabelle Moltini-Conclois, Valerie Courgnaud, Dorothée Missé, Nadine Laguette, and Karim Majzoub. Please ensure that the full contributions of each author are acknowledged in the "Add/Edit/Remove Authors" section of our submission form.

The list of CRediT author contributions may be found here: https://journals.plos.org/plosntds/s/authorship#loc-author-contributions

2) Please provide an Author Summary. This should appear in your manuscript between the Abstract (if applicable) and the Introduction, and should be 150-200 words long. The aim should be to make your findings accessible to a wide audience that includes both scientists and non-scientists. Sample summaries can be found on our website under Submission Guidelines:

https://journals.plos.org/plosntds/s/submission-guidelines#loc-parts-of-a-submission

3) We do not publish any copyright or trademark symbols that usually accompany proprietary names, eg ©,  ®, or TM  (e.g. next to drug or reagent names). Therefore please remove all instances of trademark/copyright symbols throughout the text, including:

- TM on pages: 19, and 20.

4) Please upload all main figures as separate Figure files in .tif or .eps format. For more information about how to convert and format your figure files please see our guidelines:

https://journals.plos.org/plosntds/s/figures

5) We have noticed that you have uploaded Supporting Information files, but you have not included a list of legends. Please add a full list of legends for your Supporting Information files after the references list.

6) In the online submission form, you indicated that All data are available in the manuscript and upon request.. All PLOS journals now require all data underlying the findings described in their manuscript to be freely available to other researchers, either

1. In a public repository

2. Within the manuscript itself

3. Uploaded as supplementary information.

This policy applies to all data except where public deposition would breach compliance with the protocol approved by your research ethics board. If your data cannot be made publicly available for ethical or legal reasons (e.g., public availability would compromise patient privacy), please explain your reasons by return email and your exemption request will be escalated to the editor for approval. Your exemption request will be handled independently and will not hold up the peer review process, but will need to be resolved should your manuscript be accepted for publication. One of the Editorial team will then be in touch if there are any issues.

7) Please amend your detailed Financial Disclosure statement. This is published with the article. It must therefore be completed in full sentences and contain the exact wording you wish to be published.

i) State what role the funders took in the study. If the funders had no role in your study, please state: "The funders had no role in study design, data collection and analysis, decision to publish, or preparation of the manuscript."

ii) If any authors received a salary from any of your funders, please state which authors and which funders..

If you did not receive any funding for this study, please simply state: u201cThe authors received no specific funding for this work.u201d

8) Your current Financial Disclosure states, "This work was co-funded by the European Union (ERC CoG, SENTINEL 101087092 to NL and ERC Stg DELV 101039538 to KM). The Agence National de la Recherche [AlphA to NL, DeltADAPT to KM and and ANR-10-LABX-25-01 to DM] and the Agence Nationale de Recherche sur le SIDA et les Hépatites virales (ANRS) [ECTZ117448 to NL and RJE].".However, your funding information on the submission form not indicates "ECTZ117448 ".Please indicate by return email the full and correct funding information for your study and confirm the order in which funding contributions should appear. Please be sure to indicate whether the funders played any role in the study design, data collection and analysis, decision to publish, or preparation of the manuscript.

8)PLOS now requires that authors provide the original uncropped and unadjusted images underlying all blot or gel results reported in a submission’s figures or Supporting Information files. This policy and the journal’s other requirements for blot/gel reporting and figure preparation are described in detail at https://journals.plos.org/plosone/s/figures#loc-blot-and-gel-reporting-requirements and https://journals.plos.org/plosone/s/figures#loc-preparing-figures-from-image-files.

When you submit your revised manuscript, please ensure that your figures adhere fully to these guidelines and provide the original underlying images for all blot or gel data reported in your submission. See the following link for instructions on providing the original image data: https://journals.plos.org/plosone/s/figures#loc-original-images-for-blots-and-gels.

In your cover letter, please note whether your blot/gel image data are in Supporting Information or posted at a public data repository, provide the repository URL if relevant, and provide specific details as to which raw blot/gel images, if any, are not available. Email us at customercare@plos.org if you have any questions.

9) Please send a completed 'Competing Interests' statement, including any COIs declared by your co-authors. If you have no competing interests to declare, please state "The authors have declared that no competing interests exist". Otherwise please declare all competing interests beginning with the statement "I have read the journal's policy and the authors of this manuscript have the following competing interests:"

**Reviewers' comments:**

**Key Review Criteria Required for Acceptance?**

As you describe the new analyses required for acceptance, please consider the following:

**Methods**

-Are the objectives of the study clearly articulated with a clear testable hypothesis stated?

-Is the study design appropriate to address the stated objectives?

-Is the population clearly described and appropriate for the hypothesis being tested?

-Is the sample size sufficient to ensure adequate power to address the hypothesis being tested?

-Were correct statistical analysis used to support conclusions?

-Are there concerns about ethical or regulatory requirements being met?

Reviewer #1: Methods: The study by Labadie and colleagues investigates how Tonate virus (TONV), a neglected encephalitic alphavirus of the Venezuelan equine encephalitis complex, is sensed in human cell lines and how this sensing induces both IFN-I production and subsequent IFN-I responses. Although both encephalitic and arthritogenic alphaviruses are known to trigger IFN-I responses, this has not been specifically examined for TONV.

The manuscript is well written and clearly articulated.

Reviewer #2: The study objectives are articulated to describe the TONV innate immune sensing of human cells.

The study design is appropriate to address the objectives.

The required sample size for the in vitro assays is sufficient.

The study contained statistical analysis that can support the observations.

Reviewer #3: All the components mentioned above, including the objectives, methods, and statistical analyses, are well described.

**********

**Results**

-Does the analysis presented match the analysis plan?

-Are the results clearly and completely presented?

-Are the figures (Tables, Images) of sufficient quality for clarity?

Reviewer #1: Results: The results and figures are appropriate.

Line-by-line comments:

Fig 3A. It is intriguing that MAVS protein levels appear less intense in WT cells than in the other

KO lines (except the MAVS KO). In addition, residual MAVS signal is still detected by Western

blot in the MAVS KO line and, by eye, does not differ clearly from WT. Please add densitometric

quantification and discuss in the text why residual MAVS signal may be detected in the KO line.

Fig. 3B-D show differences in viral replication, IFN-I induction, and downstream responses

between IRF3-KO and MAVS-KO cells, suggesting additional sensing pathways contributing to IFN-I induction. TLR3-TRIF signaling can activate IRF3 and has been implicated in alphavirus infection (PMID 22761364; PMID 27864552). Additional IRF3 activation through this pathway could explain the IRF3-KO versus MAVS-KO difference and is consistent with MAVS-IRF3 not being the unique or major pathway implicated on TONV-induced IFN-I production. The study would be strengthened by testing the contribution of TLR3 or, at minimum, discussing this additional possibility.

Fig. S2B. In the Results, the authors state that “We also confirm that IFN pretreatment increases

ISG levels in both cell lines”. This statement is not supported for A549 cells. Also, add statistical

analysis for this experiment.

Reviewer #2: The results presented require clear description and organization. Several sentences are redundant, and some of the sentences and citations can be placed in the Introduction and Discussion sections.

Further description of the results in the text is required. For example: in case of any significant differences (p-values) in the ISG (MX1) levels in both cell lines during the IFN-1 treatment, TONV RNA loads in the absence of IRF3 at different MOI. It would be interesting to discuss the low IFN-β production compared to WT cells upon infection at low MOI (E.g., 0.001).

Please include the legends for all the manuscript figures, as they were not found in the submission documentation. Figure legends are critical for revising the figures. Provide a color image for Figure #4, as no differences are readily visible between the non-infected and infected T98G cells

Reviewer #3: Analyses and results are clearly presented.

**********

**Conclusions**

-Are the conclusions supported by the data presented?

-Are the limitations of analysis clearly described?

-Do the authors discuss how these data can be helpful to advance our understanding of the topic under study?

-Is public health relevance addressed?

Reviewer #1: - The conclusions are supported by the data.

- The study would be strengthened by discussing the contribution of other sensors such as TLR3.

- Limitations are described appropriately

- The authors discuss both the advance in knowledge as well as public health relevance.

Reviewer #2: Conclusions are partially supported by the data presented.

The authors described limitations of the study, such as the use of two immortalized human cell lines, the exact viral RNA species recognized during TONV infection, and the contribution of the two RLRs over time.

Yes, the authors discussed how this model can be helpful for testing antivirals.

Yes, studying TONV innate immune sensing in vitro has relevance to public health.

Reviewer #3: The conclusions are well supported by the data presented, and the limitations of the study are appropriately acknowledged.

**********

**Editorial and Data Presentation Modifications?**

Use this section for editorial suggestions as well as relatively minor modifications of existing data that would enhance clarity. If the only modifications needed are minor and/or editorial, you may wish to recommend “Minor Revision” or “Accept”.

Reviewer #1: Fig. 1B. For clarity, specify the cell line used. This is not apparent from either the text or the figure.

Fig. 1D. The nature of the IFN-I reporter assay is unclear. The manuscript would benefit from one sentence in the Results stating whether this is a reporter-cell assay or a direct measurement of interferon (e.g., ELISA). This information is already in the Materials and Methods, but a brief clarification in the Results would improve readability.

Fig. 2. It is not clear from the text as written that the KO lines were generated in the glioblastoma cell line. Please clarify in the result description in which cell types the KO were made.

Reviewer #2: (No Response)

Reviewer #3: (No Response)

**********

**Summary and General Comments**

Use this section to provide overall comments, discuss strengths/weaknesses of the study, novelty, significance, general execution and scholarship. You may also include additional comments for the author, including concerns about dual publication, research ethics, or publication ethics. If requesting major revision, please articulate the new experiments that are needed.

Reviewer #1: Revision. Manuscript PNTD-D-26-01338

This study from Labadie and colleagues explores an important question in alphavirus biology: how Tonate virus (TONV), a neglected encephalitic alphavirus of the Venezuelan equine encephalitis complex, is sensed in human cells and how that sensing drives type I interferon production and downstream responses. The authors use a strong set of experimental approaches, including infection of human cell lines, genetic knockouts of RIG-I, MDA5, MAVS, and IRF3, and complementary readouts of viral replication, IFN-I induction, and ISG expression. While some weaknesses have been identified (see point-by-point comments), this study provides solid evidence that the RIG-I/MDA5–MAVS–IRF3 axis is a major pathway of TONV restriction in human cells. The work is well written, clearly articulated, and fills a genuine gap, as this question has not been specifically examined for TONV.

Comments:

Fig 3A. It is intriguing that MAVS protein levels appear less intense in WT cells than in the other KO lines (except the MAVS KO). In addition, residual MAVS signal is still detected by Western blot in the MAVS KO line and, by eye, does not differ clearly from WT. Please add densitometric quantification and discuss in the text why residual MAVS signal may be detected in the KO line.

Fig. 3B-D show differences in viral replication, IFN-I induction, and downstream responses

between IRF3-KO and MAVS-KO cells, suggesting additional sensing pathways contributing to IFN-I induction. TLR3-TRIF signaling can activate IRF3 and has been implicated in alphavirus infection (PMID 22761364; PMID 27864552). Additional IRF3 activation through this pathway could explain the IRF3-KO versus MAVS-KO difference and is consistent with MAVS-IRF3 not being the unique or major pathway implicated on TONV-induced IFN-I production. The study would be strengthened by testing the contribution of TLR3 or, at minimum, discussing this additional possibility.

Fig. S2B. In the Results, the authors state that “We also confirm that IFN pretreatment increases ISG levels in both cell lines”. This statement is not supported for A549 cells. Also, add statistical analysis for this experiment.

Minor:

• Fig. 1B. For clarity, specify the cell line used. This is not apparent from either the text or the figure.

• Fig. 1D. The nature of the IFN-I reporter assay is unclear. The manuscript would benefit from one sentence in the Results stating whether this is a reporter-cell assay or a direct measurement of interferon (e.g., ELISA). This information is already in the Materials and Methods, but a brief clarification in the Results would improve readability.

• Fig. 2. It is not clear from the text as written that the KO lines were generated in the glioblastoma cell line. Please clarify in the result description in which cell types the KO were made.

Reviewer #2: Summary

Tonate virus (TONV) is a neglected arbovirus of the Venezuelan equine encephalitis complex associated with febrile illness and severe disease. However, TONV epidemiology and diagnostics are under the umbrella of dengue disease in the Americas. The study aimed to describe the interaction between TONV and the IFN-I system in two human cell lines. T98G and A549 cells derived from human glioblastoma and lung carcinoma, respectively, were susceptible to TONV infection at different multiplicities of infection (MOI). TONV infection elicited an IFN-I response in both human cell lines. A further treatment with exogenous IFN-I inhibited TONV infectivity and viral RNA, with increased levels of certain Interferon-stimulated genes. Furthermore, TONV infection led to the accumulation of cytosolic double-stranded (ds)RNA. In vitro depletion of cellular components upstream of the IFN-I generation (RIG-I, MDA5, MAVS, but mainly IRF-3) increased viral RNA and dsRNA-positive cells. The study indicated the RIG-I/MDA5-MAVS-IRF3 axis as a major sensing pathway during TONV infection, and described for the first time a molecular characterization of TONV innate immune sensing in human cells. However, the manuscript requires more attention in the methods and results sections for further publication consideration.

Comments

• Include line numbers throughout the manuscript

• Include Figure legends

• Suggest following the research article structure: Introduction, followed by Materials and Methods, Results, and Discussion/Conclusions.

• Figure #4. Clarify the % difference in positive cells between infected and non-infected WT cells.

• Support the Discussion section by citing Figures

• Provide the passage history of the TONV CaAn 410d strain

• Justify the use of two different housekeeping genes (18S and GAPDH)

Reviewer #3: This is a well-written and well-structured manuscript describing the mechanisms underlying type I IFN inhibition following TONV infection in immune cell lines in vitro. Although the experiments were performed using immortalized cell lines, the authors conducted a comprehensive series of important experiments investigating critical components of the type I interferon pathway in immune cells.

Minor comment

As all experiments were performed using immortalized cell lines, I suggest revising the statement in the abstract that says, “…we investigated the interaction between TONV and the type I interferon (IFN-I) system in human cells.” This statement may be too broad, given that the study was conducted exclusively in immortalized cell lines. The authors may wish to specify this experimental context more clearly.

**********

PLOS authors have the option to publish the peer review history of their article (what does this mean?). If published, this will include your full peer review and any attached files.

If you choose “no”, your identity will remain anonymous but your review may still be made public.

**Do you want your identity to be public for this peer review?**  For information about this choice, including consent withdrawal, please see our Privacy Policy.

Reviewer #1: No

Reviewer #2: No

Reviewer #3: No

**Figure resubmission:**

**Reproducibility:** -->To enhance the reproducibility of your results, we recommend that authors of applicable studies deposit laboratory protocols in protocols.io, where a protocol can be assigned its own identifier (DOI) such that it can be cited independently in the future. Additionally, PLOS ONE offers an option to publish peer-reviewed clinical study protocols. Read more information on sharing protocols at https://plos.org/protocols?utm_medium=editorial-email&utm_source=authorletters&utm_campaign=protocols-->?>

Attachment Submitted filename: PNTD-D-26-01338-.pdf

# Author comment: Author response to Decision Letter 1

**DOI:** 10.1371/journal.pntd.0014559.r002

6 Oct 2026

Attachment Submitted filename: Labadie Eloiflin et al - Response to reviewers.docx

# Editor report: Decision Letter 1

**DOI:** 10.1371/journal.pntd.0014559.r003

**Authors:** Doug Brackney, David Safronetz

8 Oct 2026

Dear Dr. Majzoub,

We are pleased to inform you that your manuscript 'The RLR-MAVS-IRF3 axis activates the IFN pathway to restrict Tonate virus infection' has been provisionally accepted for publication in PLOS Neglected Tropical Diseases.

Before your manuscript can be formally accepted you will need to complete some formatting changes, which you will receive in a follow up email. A member of our team will be in touch with a set of requests.

Please note that your manuscript will not be scheduled for publication until you have made the required changes, so a swift response is appreciated.

IMPORTANT: The editorial review process is now complete. PLOS will only permit corrections to spelling, formatting or significant scientific errors from this point onwards. Requests for major changes, or any which affect the scientific understanding of your work, will cause delays to the publication date of your manuscript.

Should you, your institution's press office or the journal office choose to press release your paper, you will automatically be opted out of early publication. We ask that you notify us now if you or your institution is planning to press release the article. All press must be co-ordinated with PLOS.

Thank you again for supporting Open Access publishing; we are looking forward to publishing your work in PLOS Neglected Tropical Diseases.

Best regards,

Doug E Brackney, PhD

Academic Editor

PLOS Neglected Tropical Diseases

David Safronetz

Section Editor

PLOS Neglected Tropical Diseases

Shaden Kamhawi

co-Editor-in-Chief

PLOS Neglected Tropical Diseases

orcid.org/0000-0003-4304-636XX

Paul Brindley

co-Editor-in-Chief

PLOS Neglected Tropical Diseases

orcid.org/0000-0003-1765-0002

***********************************************************

# Editor report: Acceptance letter

**DOI:** 10.1371/journal.pntd.0014559.r004

**Authors:** Doug Brackney, David Safronetz

Dear Dr. Majzoub,

We are delighted to inform you that your manuscript, "The RLR-MAVS-IRF3 axis activates the IFN pathway to restrict Tonate virus infection," has been formally accepted for publication in PLOS Neglected Tropical Diseases.

We have now passed your article onto the PLOS Production Department who will complete the rest of the publication process. All authors will receive a confirmation email upon publication.

The corresponding author will soon be receiving a typeset proof for review, to ensure errors have not been introduced during production. Please review the PDF proof of your manuscript carefully, as this is the last chance to correct any scientific or type-setting errors. Please note that major changes, or those which affect the scientific understanding of the work, will likely cause delays to the publication date of your manuscript. Note: Proofs for Front Matter articles (Editorial, Viewpoint, Symposium, Review, etc...) are generated on a different schedule and may not be made available as quickly.

Soon after your final files are uploaded, the early version of your manuscript will be published online unless you opted out of this process. The date of the early version will be your article's publication date. The final article will be published to the same URL, and all versions of the paper will be accessible to readers.

For Research Articles, you will receive an invoice from PLOS for your publication fee after your manuscript has reached the completed accept phase. If you receive an email requesting payment before acceptance or for any other service, this may be a phishing scheme. Learn how to identify phishing emails and protect your accounts at https://explore.plos.org/phishing.

Thank you again for supporting open-access publishing; we are looking forward to publishing your work in PLOS Neglected Tropical Diseases.

Best regards,

Shaden Kamhawi

co-Editor-in-Chief

PLOS Neglected Tropical Diseases

Paul Brindley

co-Editor-in-Chief

PLOS Neglected Tropical Diseases
